# Assembly, Activation, and Helicase Actions of MCM2-7: Transition from Inactive MCM2-7 Double Hexamers to Active Replication Forks

**DOI:** 10.3390/biology13080629

**Published:** 2024-08-17

**Authors:** Zhiying You, Hisao Masai

**Affiliations:** 1 Genome Dynamics Project, Department of Basic Medical Sciences, Tokyo Metropolitan Institute of Medical Science, Setagaya-ku, Tokyo 156-8506, Japan; 2 Department of Computational Biology and Medical Sciences, Graduate School of Frontier Sciences, The University of Tokyo, Chiba 277-8561, Japan

**Keywords:** MCM2-7, Cdc45/MCM2-7/GINS (CMG), DNA helicase, Cdc7 (DDK), CDK

## Abstract

**Simple Summary:**

MCM2-7, evolutionarily conserved and essential for DNA replication, functions as the central factor for the eukaryotic replicative DNA helicase. Here, we summarize the roles of MCM2-7 in the initiation and progression of replication forks, with a particular focus on the assembly of the replication complex and its regulation. We also describe the molecular details of the steps required for the transition from the inactive MCM2-7 double hexamer to an active replication fork.

**Abstract:**

In this review, we summarize the processes of the assembly of multi-protein replisomes at the origins of replication. Replication licensing, the loading of inactive minichromosome maintenance double hexamers (dhMCM2-7) during the G1 phase, is followed by origin firing triggered by two serine–threonine kinases, Cdc7 (DDK) and CDK, leading to the assembly and activation of Cdc45/MCM2-7/GINS (CMG) helicases at the entry into the S phase and the formation of replisomes for bidirectional DNA synthesis. Biochemical and structural analyses of the recruitment of initiation or firing factors to the dhMCM2-7 for the formation of an active helicase and those of origin melting and DNA unwinding support the steric exclusion unwinding model of the CMG helicase.

## 1. Introduction

The MCM2-7 protein complex is evolutionarily conserved and essential for DNA replication. The complex consists of six subunits, MCM2 through MCM7, each of which is an AAA+ ATPase and forms a ring-shaped hexamer [1]. MCM2-7 serves as a central factor for the replicative DNA helicase that unwinds double-stranded DNA during replication. Mutations in the MCM2-7 genes induce chromosome instability, thereby crucially affecting genome integrity. Here, we will summarize the most recent findings on the assembly and activation of the eukaryotic replicative DNA helicase. The process of DNA replication initiation first involves the assembly of the pre-replication complex (pre-RC) containing the Origin Recognition Complex (ORC) and the licensing factors, Cdt1 and Cdc6, which function during G1 to load the MCM2-7 proteins onto replication origin DNA as inactive double hexamers [2]. The activation of MCM2-7 double hexamers (dhMCM2-7) into an active Cdc45/MCM2-7/GINS (CMG) helicase complex requires essential firing factors, including Treslin–MTBP (Sld3-Sld7) [metazoan (budding yeast)] complex, RecQL4, DONSON (Sld2), and TopBP1 (Dpb11), along with phosphorylation by Cdc7/Dbf4 kinase, Dbf4-dependent kinase (Cdc7/DDK), and cyclin-dependent kinase (CDK) during the G1–S transition and throughout the S phase [3,4,5]. The interplay between CDK and Cdc7 kinases regulates the efficient activation of dhMCM2-7, orchestrating bidirectional origin firing critical for maintaining genome integrity during cellular division. During replication initiation, phosphatases, including protein phosphatase 1 (PP1), PP2A, and PP4, counteract Cdc7 and CDK, balancing protein phosphorylation to control origin firing [6]. The activation of the CMG helicase requires Mcm10, an essential gene, after CMG assembly for bidirectional replication fork movement [7]. Cryo-electron microscopy (cryo-EM) structural analyses reveal MCM2-7 encircles and unwinds the leading DNA strand through coordinated ATP hydrolysis via the steric exclusion mechanism [8]. Overall, these insights underscore the coordinated actions of the replication initiation factors and CMG helicase in facilitating efficient DNA replication initiation and progression. Readers are referred to the article in the same review series by Rankins for the functions of MCM2-7 in addition to its role in DNA unwinding [9].

## 2. Discovery of the MCM2-7 Genes

Homologs of the MCM2-7 proteins are found in a wide range of organisms and are present in both archaea and eukaryotes, highlighting their conserved and vital role in genome stability during cellular proliferation. The discovery of the MCM genes marked a milestone in the study of DNA replication. Initially identified through genetic studies in the budding yeast *Saccharomyces cerevisiae* by Tye and her colleagues, mutations in the MCM2-7 genes were found to induce chromosome instability and aberrations in the maintenance of minichromosomes (plasmids) [10]. *MCM3*, *MCM2*, *Cdc46* (*MCM5*), *Cdc47* (*MCM7*), and *Cdc54* (*MCM4*) genes were identified as the responsible genes [11,12]. These mutations interact with *Cdc45* mutations, and the mutants are genetically defective in their progression through the S phase of the cell cycle [12]. *Cdc21* (*MCM4*), *Nda4* (*MCM5*), and *Mis5* (*MCM6*) mutants were isolated and analyzed in the fission yeast *Saccharomyces cerevisiae* [13,14,15,16]. Individual members of MCM2-7, including Cdc21 (MCM4) and Cdc46 (MCM5), were found to share conserved sequences and belong to a family of proteins that are involved in DNA replication and are highly conserved in evolution [12,13,17]. Using antibodies that recognize regions commonly present in MCM2-7, the MCM2-7 proteins were shown to be present in human cells [18]. Mouse P1 (MCM3), known to interact with DNA polymerase alpha protein, was identified [19]. This led to the first direct observation of a protein consistent with the behavior of a hypothesized factor that restricts chromatin replication to once per cell cycle in higher eukaryotes [19]. The concept of DNA replication licensing refers to the mechanism that restricts DNA replication to only once per cell cycle in eukaryotic cells, which was originally proposed by Blow and Laskey [20]. Later, the hypothetical “licensing factor” was identified as MCM2-7 in the laboratories of Takisawa [21], Blow [22], and Laskey [23]. In DNA replication studies using Xenopus egg extract, MCM3 or a complex containing MCM3 and two other polypeptides (MCM2 and 5) were identified as being responsible for DNA replication licensing [21,22,23].

## 3. Discovery of DNA Helicase Activity in MCM2-7

The MCM2-7 proteins contain ATP-binding motifs and were suggested to function as DNA helicases that unwind dsDNA in DNA replication [24]. However, helicase activity could not be detected in the purified fission yeast MCM2-7 heterohexamer [25]. Ishimi took advantage of histone H3/H4 columns that can bind to MCM2 to purify two complexes, a tetramer of MCM2/4/6/7 and a hexamer of MCM(4/6/7)2, from Hela cells extracts. In 1997, he reported for the first time that human hexameric MCM(4/6/7)2 has single-stranded DNA-dependent ATP hydrolysis activity and DNA helicase activity exhibiting a 3′→5′ direction [26]. Two years later, You and Ishimi purified a recombinant mouse MCM4/6/7 complex and demonstrated that hexameric MCM(4/6/7)2 possesses intrinsic DNA helicase activity [27]. The three subunits, MCM4, 6, and 7 proteins, contribute to the helicase activity of the complex by playing distinct biochemical functions [27,28]. A forked or bubble-like DNA structure is required for the formation of the double heterohexameric complex of MCM4/6/7 [29,30]. Unlike yeast where origin sequences are AT-rich, no essential or conserved sequence motifs have been identified for mammalian replication origins. However, thymine-rich single-stranded DNA on bubble or fork structures preferentially activates the ATPase and helicase activities of the MCM4/6/7 helicase. Based on these, Masai’s lab proposed a possible role for MCM4/6/7 helicase in the selection of replication initiation sites in mammalian genomes [30]. A similar outcome was observed where the unwinding activity of CMG-Mcm10 was significantly reduced on a derivative of ARS1 with decreased AT % [31]. In vitro, helicase assays indicated the intrinsic preference of MCM4/6/7 to T-rich sequences [30,32]. An alternative explanation could be that A/T base pairs are more unstable than G/C base pairs, rendering A/T-rich sequences more prone to unwinding, exposing single-stranded DNA to which MCM4/6/7 has an affinity. A study of double hexameric MCM2-7 human genome enrichment patterns supporting this proposal was published last year [33]. Mapping of the endogenous human dhMCM2-7 footprints indicates that initial open structures are distributed across the genome in large clusters aligning well with the initiation zones designed for stochastic origin firing. A recent study showed that replication initiation zones are critically confined by Cohesin-mediated loop anchors [34]. Repli-seq and optical replication mapping (ORM) methods reveal that initiation zones become more focused following the knockdown of the Cohesin unloading factor WAPL, while they become less focused in Rad21 (Cohesin) knockdown cells compared to the wild-type condition. These findings suggest that Cohesin-mediated loop anchors regulate the precise positioning of human replication origins. Interestingly, sequence composition analysis of MCM2-7-bound DNAs showed that they lack a consensus motif but are highly AT-rich. The highest AT content is located at the center of the dhMCM2-7 sites, suggesting that human MCM2-7 hexamers preferentially bind to sequences prone to unwinding to facilitate initial DNA melting. The addition of MCM2 or MCM3/5 dimers to MCM(4/6/7)2 inhibited helicase activity [27,35,36,37]. Therefore, it was thought that MCM4, 6, and 7 had a catalytic role in its helicase function, and MCM2, 3, and 5 had a regulatory role. Subsequently, the MCM2-7 heterohexamer of *S. cerevisiae* showed ATP hydrolysis activity, and the mutational analyses of the ATP-binding sites of MCM2-7 subunits revealed the regulatory roles of MCM2, 3, and 5 in the MCM2-7 helicase function [38].

## 4. MCM2-7 as a Central Factor for an Active Replicative Helicase Complex

In 2000, Blow’s laboratory prepared several MCM subcomplexes from Xenopus egg extract and clarified the assembly pathway of the MCM2-7 hexamer from subcomplexes and showed that it is the heterohexamer, rather than the subcomplex, that supports DNA replication [39]. In the same year, Labib et al. in the Diffley lab showed that all MCM2-7 proteins are not only required for the initiation but are also essential for the elongation phase of *S. cerevisiae* DNA replication [40]. These results suggest that only the full MCM2-7 heterohexamer provides replication licensing activity and functions in the initiation and progression of replication. In vivo studies of eukaryotic replicative helicases showed the importance of the heterohexameric MCM2-7 complex, whereas in vitro studies showed that helicase activity is associated with only specific subsets (hexameric MCM4/6/7 and MCM4/7) [38,41,42]. However, Bochman and Schwacha reported that the *S. cerevisiae* MCM2-7 hexamer exhibits DNA helicase activity under special conditions that contain glutamate and acetate anions in the reaction solution [43]. Nevertheless, our group was unable to detect the helicase activity of the mouse MCM2-7 complex under the same conditions. We discovered that the MCM2-7 complex has potent DNA strand annealing activity, which reanneals the unwound DNA strands and masks its intrinsic DNA helicase activity [44]. A single-molecule biochemical study that monitored the translocation of CMG and unwinding of DNA suggests that CMG exhibited not only unwinding and pausing but also a reverse motion for annealing [45]. Given that the annealing activity of MCM2-7 is inhibited by the presence of ATP and activated by ADP [44], the unwinding/reannealing may be regulated by ATP hydrolysis.

It is now well established that the MCM2-7 complex is a part of the cellular machine responsible for the unwinding of DNA during the S phase [46,47,48,49]. In one of the early reports, a CMG complex consisting of Cdc45, MCM2-7, and GINS purified from Drosophila embryo extracts exhibited DNA helicase activity under normal reaction conditions [47]. The initiation factor Cdc45 directly binds to MCM2-7 and functions in the initiation of DNA replication. GINS, a complex formed by the proteins Sld5, Psf1, Psf2, and Psf3, was discovered through yeast genetic screens aimed at the identification of novel replication factors [50]. Physical interaction between MCM2-7, Cdc45, and GINS proteins was originally shown by their co-immunoprecipitation in Xenopus egg extracts [51]. Using yeast, a protein complex containing both MCM2-7 and GINS yielded a large protein structure (>1400 KD) assembled specifically during the S phase, which is termed the “replisome progression complex” or “RPC” [46]. The main components of the RPC identified by mass spectrometry are MCM2-7, Cdc45, and GINS, along with Mrc1, Tof1, Csm3 (a fork protection complex), Ctf4, and FACT. In addition, the human CMG complex unwinds relatively long double-stranded DNA and promotes DNA synthesis by DNA polymerase ε [52]. Therefore, the CMG complex constitutes the core of an RPC and plays a pivotal role in the progression of the replisome.

## 5. Transition of the Inactive MCM2-7 Double Hexamer to an Active Replication Fork

**Figure 1:**
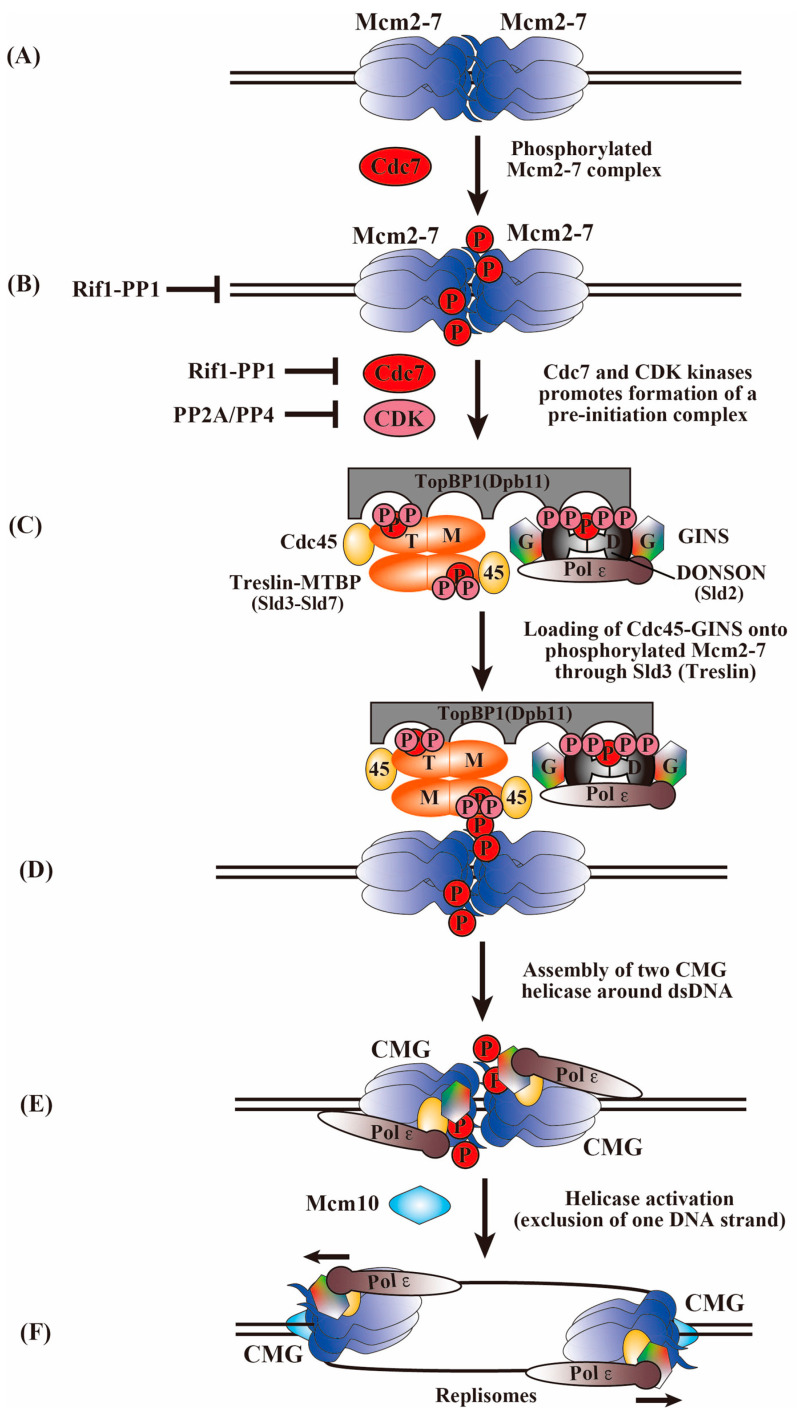
A schematic model for CMG assembly and activation. (**A**) A licensed origin on which dhMCM2-7 is loaded. MCM2-7 complexes are assembled around double-stranded DNA at replication origins during G1 phase, forming a head-to-head double hexamer that lacks helicase activity. (**B**) During the S phase, Cdc7 phosphorylates the MCM2-7 double hexamers, most notably at the N-terminal tails of MCM4, MCM6, and MCM2 subunits. Phosphorylation is reversed by PP1, which can be recruited in the vicinity of origins by Rif1 and counteracts Cdc7-mediated origin firing. Also, the phosphorylation of Sld3 and Sld2 by CDK is rapidly reversed by the actions of PP2A and PP4 phosphatases. (**C**) In *S. cerevisiae*, the phosphorylated MCM2-7 generates binding sites for Sld3, which specifically recognizes the phosphorylated double hexamers, leading to the recruitment of Cdc45. Meanwhile, CDK also phosphorylates Treslin (Sld3) of the tetrameric Treslin–MTBP (Sld3–Sld7) complex and DONSON (Sld2). Phosphorylation of Treslin (Sld3) promotes its interaction with TopBP1(Dpb11) through its C-terminal phospho-binding BRCT domains, while phosphorylated Donson (Sld2) also interacts with TopBP1 (Dpb11) through its N-terminal phospho-binding BRCT domains. Treslin–MTBP can also be phosphorylated by Cdc7 and is negatively regulated by PP1 or PP2A/PP4 protein phosphatase. (**D**) Loading of Cdc45-GINS onto phosphorylated MCM2-7 through Sld3 (Treslin). (**E**) The recruitment of Cdc45 and GINS initiates the assembly of two CMG helicase complexes. Meanwhile, the MCM2-7 rings remain encircling dsDNA, while initial open structures are generated due to the untwisting and separation of the double hexamer into two discrete parts. (**F**) Mcm10 interacts with the N-terminal homo-dimerization face of the MCM2-7 helicase and activates CMG through the ATPase function of MCM2-7, stimulating further untwisting of unwound DNA. Mcm10 protein facilitates transient opening of the MCM2-7 ring to exclude one DNA strand, thereby enabling full activation of the two helicases at both replication forks.

The basic mechanism of DNA replication is evolutionally conserved. The process of chromosomal DNA replication consists of replication initiation (origin firing) and elongation, including the unwinding of double-stranded DNA and the synthesis of a new DNA strand. The MCM2-7 proteins play important roles in each of these steps. Before replication initiation is permitted, a pre-RC containing Cdc6, Cdt1, and MCM2-7 complexes is assembled on the ORC at prospective replication initiation sites. After that, Cdc45, GINS, Treslin (Sld3, the budding yeast ortholog), DONSON/RECQL4 (Sld2), TopBP1 (Dpb11), Mcm10, etc., accumulate in a manner dependent on two kinases, CDK and Cdc7/DDK, and a pre-IC is formed, and DNA replication begins [53,54]. Two hexameric MCM2-7 helicase complexes are loaded around the origin DNA during the G1 phase as head-to-head MCM2-7 double hexamers connect via their N-terminal rings (Figure 1A) [55,56]. Inactive dhMCM2-7 is activated via association with other replication factors to form a CMG helicase for DNA unwinding (Figure 1). This process is regulated by CDK and Cdc7 during the G1–S transition and throughout the S phase [57,58]. At the replication fork, a large complex, the RPC, is formed and is responsible for the initiation, DNA chain elongation, and stable maintenance of the replication forks [59,60,61]. The initial unwinding of double-stranded DNA by two active CMG helicase complexes and firing factors allows the establishment of bidirectional replication forks [62,63].

## 6. Phosphorylation of MCM2-7 by Cdc7 and CDK Kinases for the Activation of the MCM2-7 Helicase and the Initiation of DNA Replication

The Cdc7 kinase in a complex with its activator Dbf4 [64,65] triggers the initiation of DNA replication through the phosphorylation of MCM2-7, which is highly conserved from yeast to humans [3,57]. In addition to its well-established roles in DNA replication initiation, Cdc7 is known to have diverse roles in regulating various chromosome dynamics, including recombination initiation, DNA repair, replication checkpoints, and heterochromatin formation [66]. Biochemical and genetic studies of Cdc7 and Dbf4 indicate that the MCM2 subunit is one of the most critical target proteins of Cdc7 in all eukaryotic cells [64,67,68,69,70,71] and that MCM4 and MCM6 are also phosphorylated by Cdc7 (Figure 1B) [67,72]. Genetic evidence for Cdc7-MCM2-7 interaction was provided by the isolation of the MCM5 mutant *bob1* as a suppressor of *cdc7*(ts) [65,73]. Although the MCM5 protein itself appears not to be a major target of Cdc7, Cdc7-dependent phosphorylation of the MCM2, 4, and 6 subunits may cause a conformational change in the MCM5 protein that is required for Cdc45 protein loading and helicase activation [74,75]. In budding yeast, humans, and Xenopus egg cell-free extracts, the phosphorylation of MCM2, 4, and 6 was shown to be Cdc7-dependent and facilitates its interaction with Cdc45 during the S phase [72,76,77,78]. In budding yeast, the N-terminal region of MCM4 is inhibitory for the MCM2-7 function, and Cdc7-mediated phosphorylation in this segment of MCM4 releases this inhibitory activity [72]. The amino-terminal ~110 bp segment of human MCM4 contains 12 CDK phosphorylation motifs (SP or TP), of which 6 are present as SSP, STP, or TSP amino acid residues. MCM2 and 4 are phosphorylated by CDK as well, suggesting a collaboration between CDK and Cdc7 in the phosphorylation of MCM2-7. Cdc7 requires acidic amino acids adjacent to the phosphorylation sites for phosphorylation [67]. In human MCM4, CDK phosphorylation at the second S/T facilitates subsequent DDK phosphorylation at the first S by creating an environment mimicking an acidic amino acid state. Similarly, prior CDK phosphorylation of the MCM2/4/6/7 complex facilitates Cdc7 phosphorylation of MCM2 [67]. This suggests that specific CDK phosphorylation sites on MCM4 may stimulate phosphorylation at other sites by Cdc7, consistent with a positive role for CDK phosphorylation in the MCM2-7 function [1]. However, a biochemical study shows that phosphorylation of MCM4 by CDK inhibits helicase and ssDNA-binding activities of the hexameric MCM4/6/7 [79], suggesting CDK has a negative effect on the MCM2-7 function. In vivo, S-CDK impairs MCM2-7 chromatin loading and inhibits DNA synthesis in mammalian cells [80]. Mitotic CDK prevents MCM2-7 helicase loading and promotes CMG disassembly through MCM7 ubiquitylation [81,82]. Cdc7 can also be phosphorylated by CDK in vitro, suggesting the possible regulation of Cdc7 by CDK [67]. In addition, although the N-terminal phosphorylation of MCM2, MCM4, and MCM6 may appear redundant, it plays a crucial role in DNA replication initiation. Although individual mutations in these regions do not affect replication or growth, combined mutations result in the loss of cell viability [76]. Therefore, the cooperative phosphorylation of MCM2-7 by CDK and Cdc7 may be important for replication initiation.

Cryo-EM analyses have provided structural support for the Cdc7-mediated phosphorylation of dhMCM2-7 [83,84,85]. Two Cdc7s are docked onto each of the coupled dhMCM2-7 to operate independently. The docking of Cdc7 onto the double hexamer is exclusively mediated by Dbf4, which engages with the N-terminal domain (NTD)-A of MCM2 from one hexamer and MCM4 and MCM6 from the opposite hexamer [83]. Also, a similar study suggested that Cdc7 recognizes loaded dhMCM2-7 by docking onto the N-terminal MCM2 of one hexamer and phosphorylates the MCM4 and MCM6 of the other hexamer [84,85]. This structural information supports the previous result that the N-terminal segment of MCM2 enhances the stable recruitment of Cdc7 to dhMCM2-7, thereby facilitating the Cdc7-mediated phosphorylation of MCM4 and 6, leading to subsequent origin activation [86]. All these observations have important implications for the mechanism of activation of dhMCM2-7 and bidirectional origin firing by phosphorylation.

## 7. Phosphorylation of Firing Factors by Cdc7 and CDK Kinases for Activation of CMG Assembly

The assembly of a stable CMG helicase requires the participation of several essential firing factors. Reconstitution experiments of origin firing with purified budding yeast proteins have identified the minimal set of firing factors required, including CDK, Cdc7, Sld3/7, Cdc45, Sld2, GINS, Dpb11, DNA polymerase ε, and Mcm10 [59]. CDK phosphorylates its targets, Sld2 and Sld3, enabling their interaction with Dpb11 BRCT domains to recruit GINS and Pol ε during CMG assembly (Figure 1C) [87,88,89,90,91,92]. Simultaneously, the phosphorylation of the N-terminal tails of MCM4 and MCM6 by Cdc7 serves as docking sites for Sld3, facilitating the loading of Cdc45 onto the dhMCM2-7 via the Sld3-Cdc45-Sld7 complex (Figure 1D) [93,94,95]. In a manner strongly dependent on both kinases and the firing factors, Cdc45 and GINS are recruited to the core structured region of MCM2-7 to form a pair of CMG complexes, an active eukaryotic replicative helicase [47,49,96,97,98]. Single-molecule biochemical assays show that Cdc45 and GINS were recruited to loaded MCM2-7 stepwise and in a manner dependent on Sld3/7 and Cdc7. The assembly proceeds in two stages. First, Cdc45 and GINS are recruited to the N-terminal tails of MCM2-7 (the formation of CtG), which is subsequently converted to the functional CMG helicase [99]. Cdc45 is sequentially recruited to Cdc7-phosphorylated MCM4 and MCM6 tails, and this step is required for recruiting GINS. Importantly, Cdc7 levels modulate the number of Cdc45 and GINS binding events to individual dhMCM2-7, thereby controlling the frequency of the final CMG formation and origin activation.

There is a debate about the hierarchy between S-CDK and Cdc7. It was suggested that the prior phosphorylation of MCM2-7 by S-phase cyclin-dependent kinase is required for Cdc7-mediated phosphorylation [67,100]. It was also reported that Cdc7 drives the recruitment of the Cdc45 replication initiation factor to origins before S-CDK action [101]. On the other hand, it was reported that Cdc7 can phosphorylate MCM2-7 either before or after CDK activation, and the order of kinase action does not affect the replication efficiency [59]. Unexpectedly, the deletion of Mrc1 or Rif1 restores DNA replication in Hsk1(Cdc7)-null cells and bypasses the functions of Cdc7 for the initiation of DNA replication [102,103]. Furthermore, growth at a high temperature permits the growth of *hsk1*-null fission yeast cells. These observations indicate that Cdc7 can be bypassed by different genetic backgrounds or in specific growth conditions. More recent studies have shown that by the acute depletion of the Cdc7 protein using the auxin-induced degradation (AID) system, the loss of Cdc7 can be tolerated in some types of cancer cells [104], suggesting that the Cdc7 functions are dispensable for cell division and can be replaced by CDK in human cells. Cdk1 (M-CDK) remained active during the G1/S transition, and Cdk1 levels were shown to increase upon Cdc7 inhibition. These findings suggest that Cdc7 and Cdk1 collaborate and can independently promote G1/S transition by phosphorylating different MCM2-7 residues, and the phosphorylation of MCM2-7 by either Cdc7 or Cdk1 is adequate for S-phase entry. Alternatively, increased CDK activity may lead to the functional inactivation of Rif1 in recruiting the PP1 phosphatase, and MCM2-7 may be maintained in a phosphorylated state (see Section 7). In *S. cerevisiae*, *mcm5-bob1* bypassed the requirement for Cdc7 in cell division [73,74]. Taken together, these different conclusions suggest that CDK and Cdc7 may not have a strict order of operations for origin firing or that the order of actions may vary, depending on cellular physiology or cell types. Regardless, it is clear that the cooperation between the two kinases is critical for efficient genome replication.

## 8. Roles of Phosphatases in the Control of Replication Initiation

Recent studies have revealed the important roles of specific dephosphorylation events in replication regulation. Phosphatases that can counteract the action of Cdc7 and CDK include PP1, PP2A, and PP4. Cdc7-dependent phosphorylation of chromatin-associated dhMCM2-7 is reversed by PP1 targeted to chromatin by Rif1, which has been shown to play crucial roles in determining replication timing in yeast, humans, and Xenopus egg extract [78,102,105,106,107]. The PP1/Rif1 complex also reverses CDK-mediated phosphorylation to protect the ORC1 protein from proteasomal degradation, thereby promoting MCM2-7 loading during the G1 phase [106]. PP1 was discovered to reverse Cdc7-mediated phosphorylation of Treslin and inhibit the interaction between Treslin–MTBP and TopBP1 [108]. PP2A/PP4 reverses the CDK-mediated phosphorylation of Sld3 and Sld2, which is crucial for genome-wide origin firing, pre-IC formation at origins, and viability [109]. Therefore, the balance between Cdc7 and PP1 or CDK and PP2A/PP4 activities must be coordinated to control the origin activation through the regulation of the Treslin–MTBP–TopBP1 complex formation [108,109] (Figure 1B). During the establishment of the pre-IC at the origin, phosphatases play a crucial role in maintaining the appropriate phosphorylation levels of the key proteins [110,111,112]. At initiation, the coordination between Cdc7 and PP1 is essential for regulating the phosphorylation level of dhMCM2-7, modulating the recruitment of replisome factors by the initiation factors Sld3/Sld7, and regulating the replication timing program. Additionally, CDK, in conjunction with PP2A/PP4, modulates the phosphorylation status of Sld3, Sld2, and Dpb11, providing another layer of regulation to control replication initiation. Phosphatases coordinate appropriate and timely kinase responses, which are critical for regulating multiple aspects of DNA replication [6]. Phosphatase-dependent feedback loops ensure that replication is initiated accurately and efficiently, thereby preventing re-replication.

## 9. Factors Required for CMG Helicase Activation during DNA Replication Initiation

As mentioned previously, a network of interactions between Sld3/Sld7, Dpb11, Sld2, and GINS is required for the initial recruitment of GINS and Cdc45 to form CMG in yeast [89,90,91]. The functional vertebrate orthologs, TOPBP1 (Dpb11), Treslin (Sld3), MTBP (Sld7), and the recently identified DONSON (Sld2) interact with each other like their yeast orthologs [113,114,115]. Treslin or MTBP depletion inhibits DNA replication by preventing the assembly of the CMG helicase during origin firing [113,114]. The mutation of Treslin’s conserved phosphorylation sites in human cells inhibits the formation of the Treslin–MTBP–TopBP1 complex. Conversely, cells with a phosphomimic Treslin mutant exhibit accelerated replication and a shorter S phase [113,114,115]. Phosphorylation of Treslin by CDK is essential for the interaction between Treslin–MTBP and TopBP1 and, accordingly, for supporting DNA replication in human cells [92,115]. Additionally, as in yeast [93], the Cdc7 activity increases and reinforces the interaction between Treslin–MTBP and licensed dhMCM2-7, working in conjunction with the CDK activity that promotes the interaction of Treslin–MTBP with TopBP1 [108]. Taken together, these findings suggest that Treslin–MTBP could be significant targets of Cdc7, and Cdc7 collaborates with CDKs to control the Treslin–MTBP function, which could play a critical role in selecting origins for initiation (Figure 1C).

Similar to Sld2 in yeast, DONSON forms a complex with GINS, TOPBP1, and Pol ε necessary for delivering the GINS complex to the MCM2-7 complex and initiating DNA replication, although it does not share any amino acid sequence similarity with Sld2 [116,117,118,119]. The depletion of DONSON leads to the disappearance of the CMG helicase from the S-phase cells, suggesting that DONSON is essential for CMG assembly during the S phase [116,117,119,120]. During CMG helicase assembly, DONSON, existing as a dimer, interacts with the BRCT3 domain of TopBP1 and is essential for placing the GINS complex onto the MCM2–7 helicase via its interactions with the AAA+ domain of MCM3 and the Sld5 subunit of GINS [116,117,118] (Figure 1C).

In contrast, the RecQ-like helicase (RecQL4) was initially proposed as the vertebrate ortholog of Sld2 due to its limited sequence homology at the N-terminus. However, the current evidence suggests that DONSON is the functional ortholog of Sld2. There is no clear evidence that RecQL4 is required for CMG loading. Analysis of DNA replication in RecQL4 knockout cells indicates that RecQL4 is not essential for origin firing [121]. Instead, RecQL4 has been shown to contribute to Pol α loading during replication initiation [122,123]. Recent single-molecule studies demonstrate that RecQL4 and DONSON have distinct roles in higher eukaryotes. RecQL4 does not function as a scaffold for GINS dimerization or facilitate CMG assembly but instead promotes the dissociation of DONSON from CMG [5].

DONSON is required for recruiting both GINS and Cdc45 to licensed origins in vertebrates [116,117,119,120]. DONSON binds to Cdc45 [117], and the depletion or degradation of DONSON impairs the chromatin association of Cdc45 [117,119,120,124]. In contrast, in *C. elegans*, the depletion of DONSON disrupts the recruitment of GINS to the origin but not that of Cdc45 during CMG assembly, whilst the chromatin loading of Cdc45 requires Treslin and TopBP1 in the early S phase [118]. Despite some apparent variation in DONSON functions during eukaryotic evolution, the TopBP1-dependent association of DONSON with the pre-IC is required for GINS and Cdc45 assembly on dhMCM2-7. Importantly, both S-CDK and Cdc7 are required for DONSON chromatin binding [116,117,119]. Therefore, upon S-CDK activation, both TopBP1 and DONSON may be recruited to phosphorylated Treslin/MTBP at the dhMCM2-7 (Figure 1D). Recent studies reveal that GINS directly interacts with TopBP1, which hinders the binding of Pol ε to GINS within MCM2-7/Cdc45. This interaction suggests a complex process involving the recruitment of Pol ε, the displacement of TopBP1, and the integration of GINS as a replicative helicase during the initiation of DNA replication [125]. The collective presence of these initiation factors enables the stable recruitment of Cdc45 and GINS into a CMG complex, leading to helicase activation (Figure 1E). Cryo-EM of the double CMG-DONSON (dCMGDo) structure demonstrates that the double CMG (dCMG) appears to be captured by the engagement of dimeric DONSON, with each DONSON protomer contacting with one CMG complex [118,124]. Patient-derived mutations that impair DONSON dimerization hinder DNA replication [124], indicating that the symmetrical engagement of two DONSON protomers with MCM3 and GINS is necessary for the initial activation of dhMCM2-7, thereby preparing for the rotation of the MCM2-7 rings. The transition from the double complex dCMG to dCMGDo involves the two MCM2-7 rings rotating clockwise relative to each other, causing a shift in the position of the central pore. As a result, the DNA untwists at the dimerization interface, preparing for the establishment of the replication fork.

Unlike the aforementioned initiation factors required for CMG assembly, Mcm10 is dispensable for assembly but activates the CMG helicase for bidirectional unwinding after its assembly at the origin [61,126,127,128,129,130]. Following the degradation of Mcm10, essential helicase subunits are still recruited to MCM2-7, but origin unwinding is blocked [126,127,128]. In addition, mutations in the conserved zinc finger of self-interacting Mcm10 abolish the chromatin association of RPA following loading of the CMG components [127,131]. These findings suggest that Mcm10 plays a novel essential role during activation of the CMG helicase at DNA replication origins through its zinc finger. In vitro, the Mcm10 protein forms a stoichiometric complex with CMG and stimulates its helicase activity. It increases the helicase processivity on the leading strand only in the presence of a trapping oligo due to its DNA annealing activity [129,132]. The DNA annealing function of Mcm10 may play a role in blocking fork regression, thereby protecting active forks from reversing. In addition to the stimulation of initial DNA unwinding, Mcm10 promotes replication elongation both in vivo and in vitro [61]. The extent of duplex unwinding by the two head-to-head assemblies of CMG alone is limited; however, the unwinding activity is stimulated by Mcm10 (Figure 1F) [31]. Indeed, CMG is activated by Mcm10 through its ATP hydrolysis to stimulate MCM2-7 hexamer-mediated further untwisting of unwound DNA [130]. ssDNA is exposed as two CMGs continue tracking in opposite directions. The most recent structural analysis indicates that Mcm10 plays a critical role in splitting the double CMG-Pol ε (dCMGE) complex by interacting with the N-terminal homo-dimerization face of the MCM2-7 helicase. This CMGE–Mcm10 complex initiates DNA unwinding from the N-terminal side of MCM, narrowing the hexamer channel and facilitating the ejection of the lagging-strand DNA [7]. Accordingly, Mcm10 is responsible for the work conducted by the two CMG motors at the origin into productive unwinding, thereby facilitating bidirectional DNA replication. In summary, the recruitment of these initiation factors to the inactive MCM2-7 complex takes place at origins by the actions of both Cdc7 and CDK and results in the activation of the replicative CMG helicase, which allows bidirectional replication to occur.

## 10. A Structural Perspective on the CMG Activation Mechanism

**Figure 2:**
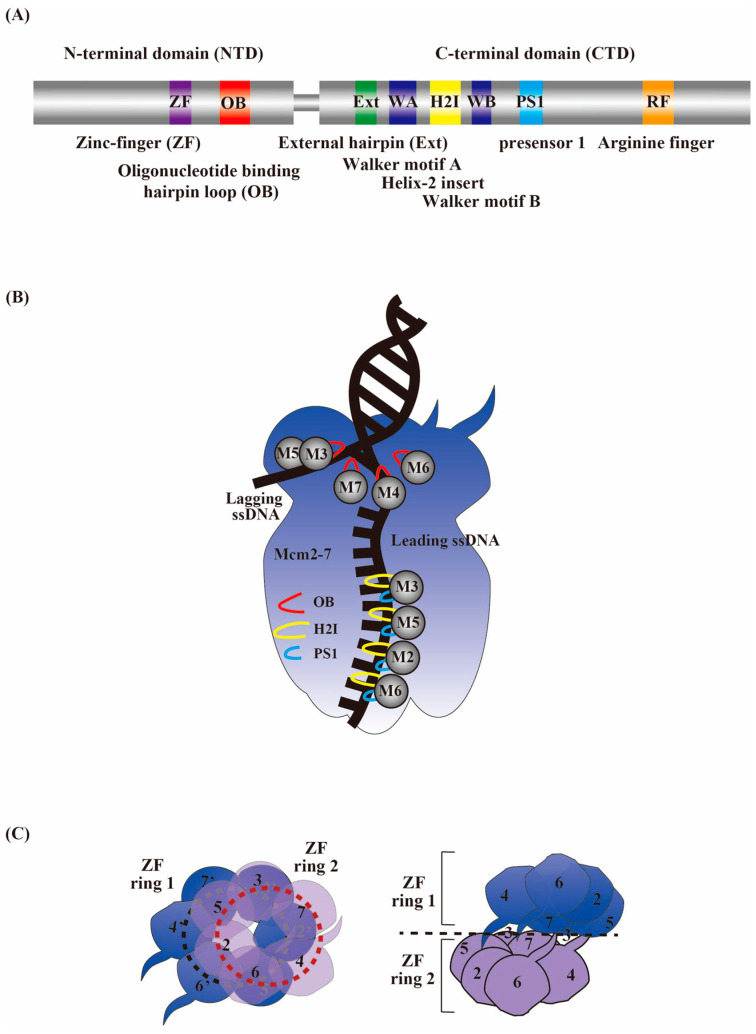
Structure of MCM2-7 and DNA unwinding in a steric exclusion mode. (**A**) A cartoon of the general domain structure of a typical MCM2-7 protein, highlighting the OB hairpin loop in the N-terminal domain and the Ext, H2I, and PS1 hairpin loops in the C-terminal domain. ZF, zinc finger domain; OB, oligonucleotide-binding hairpin; Ext, external hairpin; WA, Walker A motif; H2I, helix 2 insert hairpin; WB, Walker B motif; PS1, presensor 1 hairpin; RF, arginine finger motif. (**B**) A steric exclusion model of DNA unwinding by CMG helicase. The oligonucleotide-binding hairpin loops (OB) in the N-tiers of MCM7, 4, and 6 form a block for incoming lagging strand DNA at the lower edge of a putative DNA channel, and the OB hairpin loop of MCM3 forms an upper wall of the channel. The MCM7 OB loop appears to function as a strand separation pin, inserted at the fork junction of the two strands. It is likely that the displaced ssDNA exits between the zinc finger domains of MCM3 and MCM5 at the N-tier surface. The presensor 1 (PS1) hairpin and helix 2 insertion (H2I) loops in the C-tier motor domains of four adjacent MCM2-7 subunits (MCM3, 5, 2, and 6) are engaged with the spiral ssDNA and pull the leading strand DNA from the N-tier for the steric exclusion process of DNA unwinding. (**C**) The double CMG formation results in a constricted double hexamer interface in the N-tier. In the N-tier of MCM2-7, the channel opening tightens at the hexamer interface by the offset configuration of the two ZF rings, encircled by six hairpin loops from the oligonucleotide-binding domain (OB) of the MCM2-7 subunits, creating a narrow diameter space. This constrained DNA-binding channel ensures the firm grasping of origin DNA by the human dhMCM2-7. Top (**left**) and side (**right**) views of the ZFs of MCM2-7 at the hexamer interface.

Research on eukaryotic replisomes has made remarkable progress in recent years through the in vitro reconstitution of DNA replication systems, including single-molecular analysis [45,59,60,101,133,134,135] and the determination of the complex structures by cryo-EM [33,63,97,136,137,138,139,140,141]. The currently accepted view indicates that the hexameric helicase unwinds DNA by steric exclusion, in which the helicase encircles the tracking strand only and excludes the other strand from the ring during translocation (Figure 2B). Biochemical experiments using strand-specific streptavidin blocks show that CMG functions by encircling the leading strand [142,143].

Eukaryotic replicative CMG helicases, composed of MCM2-7 and the cofactors Cdc45 and GINS, bind at the interface between MCM2 and MCM5 within the N-layer, and this binding increases the stability and activity of CMG. Structural analyses show that dhMCM2-7 are linked together to form a head-to-head double hexamer connected via their N-terminal interfaces in a tilted and twisted manner [55,144,145,146,147]. Cryo-EM analyses of each MCM2-7 subunit of *S. cerevisiae* reveal that hairpin structures, such as the External hairpin (Ext), DNA-binding presensor 1 (PS1), and helix 2 insertion (H2I) β-hairpin loops, are found in each MCM, similar to archaeal MCMs (Figure 2A) [139,145,148]. The PS1 and H2I loops in the C-terminal tier AAA+ ATPase motor domain pull the DNA through the diversion tunnel in the N-tier containing zinc-binding domains for the steric exclusion process of DNA unwinding (Figure 2B) [138,149]. As a result of the hydrolysis of bound ATP, the hairpin structure changes, causing the movement of MCM2-7 on DNA. The ATP-dependent DNA translocation of both MCM2-7 and CMG complexes has been studied in detail [33,84,96,140,144,147,149,150]. The asymmetry in the ATP site action in CMG-DNA shows that the four neighboring MCM2-7 subunits 3, 5, 2, and 6 are engaged with a segment of single-stranded DNA via identical interactions through their PS1 and H2I loops (Figure 2B) [63,136,149,150]. In the CMG–DNA structure, three out of the six ATP-binding sites (specifically, the MCM6/2, MCM2/5, and MCM5/3 sites) are occupied by a nucleotide. The subunits at these sites also bind ssDNA, indicating structural crosstalk between nucleotide binding and ssDNA binding. On the other hand, the structural analysis of Drosophila CMG identifies three major different structures for the binding of MCM2-7 to DNA in the translocating CMG. In addition to MCM 3/5/2/6, which circles the single-stranded DNA, MCM2/6/4/7 or MCM6/4/7/3 is also able to bind to DNA by encircling single-stranded DNA [140]. Thus, not all the ATPs at the six ATP-binding sites are hydrolyzed, and ssDNA is engaged with four adjacent ATPase subunits of MCM2-7 through a series of PS1 and H2I loops arranged in a right-handed staircase spiral, establishing the C-tier staircase structure of MCM2-7. The observed asymmetric rotary mechanism of the MCM subunits [140] may provide an explanation for the differential ATP-binding site requirements across different hexamer interfaces and the differential functional significance of the varied MCM2-7 subunits. Indeed, ATP is located at MCM2/6, MCM3/5, and MCM4/7 in the human dhMCM2-7 [33], while yeast CMGE exhibits ATP binding at MCM2/5, MCM5/3, and MCM3/7 [63]. This binding pattern differs from yeast dhMCM2-7, where ATP binds only at the MCM2/6 site [84,144]. Regardless of the differences in the nucleotide-binding pattern, the ATP-dependent translocase functions on the spirally configured leading strand within a C-terminal tier of the AAA+ ATPase motor ring, suggesting common functional characteristics.

Mutations in the zinc finger domains (ZFs) of MCM4 increased the activity of the mouse hexameric MCM4/6/7 helicase, indicating that the ZFs in the N-terminal region of MCM were not essential for helicase activity [28], while ZF was necessary for archaeal MCM helicase activity [151]. This motif was found to be required for the dimerization of hexamers in the archaeal bacterium *Methanobacterium thermoautotrophicum* MCM [152] and the growth of yeast [153]. The central DNA channel constricts at the hexamer interface by an offset arrangement of two zinc finger rings surrounded by six hairpin loops from the oligonucleotide-binding (OB) domain of the MCM2-7 subunit, ensuring that human dhMCM2-7 captures the DNA strand (Figure 2C) [33]. The OB hairpin loops in the N-tiers of MCM 7, 4, and 6 form a lower block for incoming lagging strand DNA, and the OB hairpin loop of MCM3 forms an upper wall. The OB loop of MCM7 appears to function as a separation pin, inserting into the two strands at the fork junction. It is likely that the displaced ssDNA exits between the zinc finger domains of MCM3 and MCM5 at the N-tier surface in yeast [138,149]. Studies on human dhMCM2-7 and yeast CMGE have revealed an initial open DNA structure at the inter-hexamer in the surface [33,63], although the structure of the yeast dhMCM2-7 showed no melting of the bound duplex DNA at the hexamer junction [144,147,150]. The determined high-resolution structure showed that the interface of the two hexamers is twisted and offset to form a narrow central channel, which can cause deformation of the DNA trapped at the hexamer junction. At the interface of the human dhMCM2-7, two pairs of ZFs from MCM5 and MCM2 are in direct contact with melted DNA, assisted by MCM3’s ZFs [33]. During CMG formation, the tight ZF interface of the double hexamer is disrupted, leaving one MCM2-7 subunit tethered, resulting in the formation of a splayed dimer and exposing duplex DNA [63].

## 11. A Structural Perspective on the Core Replisome Cooperative Activation Mechanism

Structural analysis supports the steric exclusion model and reveals that the CMG helicase facilitates the initial melting and subsequent separation of the origin DNA strand through coordinated ATP hydrolysis action either locally at the hexamer junction or progressively within the C-terminal ring of the MCM2-7 complexes via a rotary mechanism. That is why two opposite-facing CMG helicases provide the motors for unwinding dsDNA, which can be achieved in biochemical experiments even if only the CMG protein is used [31,154]. However, the ability of CMG alone to unwind duplex DNA is limited and is greatly stimulated by the addition of Mcm10, which enables CMG to bypass blocks on the lagging strand [129].

In addition to CMG structural studies, the cryo-EM structure of the eukaryotic replisome containing the heterotrimeric fork protection complex (Csm3/Tof1 and Mrc1) and Ctf4 was also determined [138]. Csm3/Tof1 is located in the N-tier of CMG at the front of the replisome and contacts the adjacent MCM2, 6, 4, and 7 subunits, where it grabs duplex DNA. Csm3/Tof1 gripping dsDNA is also capable of monitoring structural perturbations on the DNA template in advance of CMG, which might be important for fork stabilization. This is consistent with the finding that Tof1 and Mrc1 can act to arrest replication forks upon DNA damage [155]. Moreover, the structure of leading strand polymerase Pol ε coupled to the replisome showed that Pol ε is cycling on and off the MCM2-7 ring in coordination with DNA translocation by CMG through Psf1 acting as a hinge [156]. Analysis of the cryo-EM structure of the human replisome containing CMG, pol ε, and four accessory factors (Tim–Tipin, Claspin, and And-1) yielded an overall architecture similar to that of the yeast replisome [136]. Fission yeast Mrc1/Claspin acts with MCM2 in the recycling of parental histone to the lagging strand via the co-chaperoning of H3-H4 tetramers [157], similar to a recent structural study showing parental histone recycling via MCM2–Tof1 coupling [158]. These results suggest that the fork protection complex may be located at the front of the MCM2-7 complex and function in parental histone recycling at replication forks. Mrc1/Claspin is known to be a target of Cdc7 kinase and plays important roles in replication fork progression, initiation, and cellular responses to replication stress. Claspin facilitates initiation by recruiting Cdc7 and thereby promoting MCM phosphorylation, notably in non-cancer cells [159,160]. The structures of yeast and human replisomes reveal that only short helical segments of Mrc1/Claspin can be detected, suggesting that Claspin is generally highly disordered over the entire molecule. It may adopt an extended and flexible configuration spanning one side of the replisome, with its N-terminal (site #1, 284–319) and two central segments (site #2, 525–540 and site #3, 592–618) interacting with Tim, MCM6, and MCM2, respectively [136]. Since the N-terminal half of Mrc1 interacts with the catalytic domain of Pol ε [161], Mrc1 might tether the flexible catalytic domain of Pol ε to this region of CMG to facilitate optimal helicase–polymerase coupling [162]. In the biochemical reconstitution assay of DNA replication, the DNA chain elongation rate is very slow with the minimal replisome [59], whereas the presence of Mrc1 and Csm3/Tof1 and leading-strand synthesis by Pol ε, together with PCNA, turn the replisome into rapid and efficient machinery [163]. Taken together, to drive replication initiation and maintain efficient fork progression, cooperative actions in the replisome centered on the CMG helicase are vitally important.
